# Mapping barriers and intervention activities to behaviour change theory for Mobilization of Vulnerable Elders in Ontario (MOVE ON), a multi-site implementation intervention in acute care hospitals

**DOI:** 10.1186/s13012-014-0160-6

**Published:** 2014-10-30

**Authors:** Julia E Moore, Alekhya Mascarenhas, Christine Marquez, Ummukulthum Almaawiy, Wai-Hin Chan, Jennifer D’Souza, Barbara Liu, Sharon E Straus, The MOVE ON Team

**Affiliations:** Li Ka Shing Knowledge Institute, St. Michael’s Hospital, 30 Bond Street, Toronto, ON M5B 1 W8 Canada; Regional Geriatric Program of Toronto, Sunnybrook Health Sciences Centre, 2075 Bayview Avenue, Toronto, ON M4N 3 M5 Canada; Department of Medicine, Faculty of Medicine, University of Toronto, 1 King’s College Circle, Medical Sciences Building, Toronto, ON M5S 1A8 Canada

**Keywords:** Mobilization, Frail, Older adult, Hospital, Adaptations, Barriers, Tailored interventions

## Abstract

**Background:**

As evidence-informed implementation interventions spread, they need to be tailored to address the unique needs of each setting, and this process should be well documented to facilitate replication. To facilitate the spread of the Mobilization of Vulnerable Elders in Ontario (MOVE ON) intervention, the aim of the current study is to develop a mapping guide that links identified barriers and intervention activities to behaviour change theory.

**Methods:**

Focus groups were conducted with front line health-care professionals to identify perceived barriers to implementation of an early mobilization intervention targeted to hospitalized older adults. Participating units then used or adapted intervention activities from an existing menu or developed new activities to facilitate early mobilization. A thematic analysis was performed on the focus group data, emphasizing concepts related to barriers to behaviour change. A behaviour change theory, the ‘capability, opportunity, motivation-behaviour (COM-B) system’, was used as a taxonomy to map the identified barriers to their root causes. We also mapped the behaviour constructs and intervention activities to overcome these.

**Results:**

A total of 46 focus groups were conducted across 26 hospital inpatient units in Ontario, Canada, with 261 participants. The barriers were conceptualized at three levels: health-care provider (HCP), patient, and unit. Commonly mentioned barriers were time constraints and workload (HCP), patient clinical acuity and their perceived ‘sick role’ (patient), and lack of proper equipment and human resources (unit level). Thirty intervention activities to facilitate early mobilization of older adults were implemented across hospitals; examples of unit-developed intervention activities include the ‘mobility clock’ communication tool and the use of staff champions. A mapping guide was created with barriers and intervention activities matched though the lens of the COM-B system.

**Conclusions:**

We used a systematic approach to develop a guide, which maps barriers, intervention activities, and behaviour change constructs in order to tailor an implementation intervention to the local context. This approach allows implementers to identify potential strategies to overcome local-level barriers and to document adaptations.

**Electronic supplementary material:**

The online version of this article (doi:10.1186/s13012-014-0160-6) contains supplementary material, which is available to authorized users.

## Background

Closing the gap between research evidence and practice in health care requires consideration of many factors, including stakeholder engagement, context, determinants of research uptake, and development and evaluation of the implementation intervention. In particular, context has a key role in the success or failure of implementation interventions [1-6]. Assessing context involves identifying and examining factors such as the setting and culture [7,8]. These factors can then be taken into account when tailoring the implementation intervention [5,9]. Some evidence suggests that tailored interventions are effective in improving professional practice [10,11]. For example, in a hospital setting, organizational culture and the administrative philosophy may influence the working environment within care units, which may in turn affect the attitudes of front-line clinicians, thereby influencing patient outcomes. These institution-level factors must therefore be considered when tailoring (or adapting) an evidence-based intervention for implementation. Furthermore, if multiple sites are involved, the intervention must be individually adapted to the context of each site [12]. For example, factors such as access to relevant resources (i.e. equipment, number of staff, and staff qualifications), the level of staff buy-in, and the rate of staff turnover may vary across sites, and the implementation approach must be customized accordingly for each site. Such tailoring can be arduous, given the potential for obstacles within the levels of health-care delivery (patient, health-care provider, organization, policy) at each site [6,13]. Further complicating this issue is research evidence demonstrating more favourable outcomes for tailored interventions that maintained their core components [14,15]. As such, although it is necessary to tailor interventions, it is also imperative that the intervention’s main objectives remain grounded in its original evidence base.

Even when contextual factors are well understood, it can be challenging to use them in tailoring the implementation intervention. Tailoring does not always involve a systematic, evidence-based approach, and the process often goes unmonitored and undocumented [8,16]. Various approaches for adapting interventions have been described [8,17,18], but there is a paucity of literature on how specific adaptations can be made to address barriers and facilitators to intervention success across different contexts [17]. More information is needed on how to develop approaches to modify interventions, so that these processes can be validated by researchers and replicated by those implementing the interventions in other contexts.

This study illustrates the development of a systematic approach to intervention adaptation, based on the example of a multicomponent implementation intervention that was delivered across 14 hospitals in Ontario, Canada. Specifically, we describe the use of a behaviour change theory to create a reference guide for the systematic adaptation of an early mobilization intervention for elderly patients across multiple hospitals. Mobilization of Vulnerable Elders in Ontario (MOVE ON) is an evidence-informed quality improvement intervention designed to promote early mobilization and prevent functional decline in older patients admitted to hospitals [19]. We proposed a multicomponent inter-professional strategy to promote mobilization, which was tailored to the context of each participating hospital. Implementation of this mobilization strategy was guided by the knowledge-to-action cycle [20]. The core components of the intervention included completing a mobility assessment and mobility care plan within 24 h of admission of any patient aged 65 years or older, mobilizing patients at least three times per day, and ensuring that mobilization was scaled and progressive. Early mobilization strategies using these recommendations have demonstrated benefits on length of stay, duration of delirium, depression, and discharge to home for older patients admitted to the hospital [21-23]. Sites were then encouraged to tailor intervention activities to the local context, without changing these core recommendations. Through focus groups with front-line clinical staff at each hospital, we identified the barriers to implementing an early mobilization strategy. The focus groups allowed hospital unit staff members to think about appropriate intervention adaptations at their respective sites. The staff had the option of using previously developed implementation intervention activities, adapting these activities, or developing new activities in their unit. All sites were required to provide inter-professional staff education on early mobilization, either in person or online; all additional activities were optional. The intervention was evaluated using an interrupted time series design, and the primary outcome was patient mobilization. Details of the methods were presented previously [19].

Before the MOVE ON intervention was rolled out across the province, a pilot study of the intervention—called Mobilization of Vulnerable Elders in Toronto (MOVE iT)—was conducted on units in four hospitals in Toronto, Canada. This pilot study helped us to understand the context for implementation and directly informed the implementation of MOVE ON. For example, through MOVE iT, we determined that implementation coaches would be useful to help in tailoring the implementation for each hospital unit. One of the coaches’ roles was to advise hospitals on how to identify barriers and facilitators and then to tailor the intervention to these contextual factors. Despite this support, there were no systematic, formally documented approaches to tailoring the intervention at each site. After MOVE ON was implemented in 14 hospitals, funding was received to expand the implementation intervention to seven more sites. For the launch of the third round of this intervention—called MOVE ON + —we developed a mapping guide for decision making about adaptations, to ensure that modifications to the intervention are documented and that they are informed by evidence-based behaviour change theories. The aim of the current study is to develop a mapping guide that maps identified barriers and intervention activities to behaviour change theory; the purpose of the mapping guide is to aid sites in selecting intervention activities that appropriately address context-specific barriers. To develop the guide, we used the ‘capability, opportunity, motivation-behaviour (COM-B) system’ of Michie and colleagues for understanding behaviour change, which is inspired by the findings from a consensus meeting of behavioural theorists and consideration of several theories. This system contains three main factors: capability, opportunity, and motivation [24]. *Capability* is defined as the individual’s psychological and physical capacity to perform particular behaviours or activities, including having the relevant knowledge and skills. *Opportunity* refers to factors external to the individual, such as the social milieu or physical environment, which allow or prevent a behaviour. *Motivation* refers to brain processes that energize and direct behaviour, such as habitual responses, emotional responses, and analytical decision making. These brain processes may be reflective, such as making plans or performing evaluations, or they may be automatic, involving emotions and impulses. To influence behaviour, an intervention activity might target one or more factors within this system. Therefore, when designing or adapting an intervention, it is important to consider which factors need to be changed to achieve the desired behavioural outcome. The mapping guide created during this project stimulates implementers to think about barriers to behaviour change in light of the complex processes within the COM-B system and to choose intervention activities that will target one or more of these factors.

## Methods

### Phase 1: create menu of suggested intervention activities based on MOVE iT data

Before the implementation of MOVE ON, the coaches—in collaboration with knowledge translation experts in the central MOVE ON Team—created a list (‘menu’) of suggested intervention activities to address specific barriers to the three recommendations identified during focus groups in the MOVE iT pilot study. For example, at the level of health-care providers, the greatest perceived barrier was insufficient time to devote to mobilization amidst a heavy workload and competing priorities. Teamwork and cooperation among health-care providers were cited as potential facilitators to overcome this barrier. To address this challenge and leverage recommended facilitators, one of the suggested intervention activities from coaches was to implement ‘huddles’, quick, stand-up, interdisciplinary team meetings held at nursing stations, with the aim of quickly reviewing progress and sharing successes or strategizing about immediate challenges. Hospitals were encouraged to use the ‘menu’ of suggested intervention activities as is, to adapt them to the local context or to create new intervention activities designed to target barriers identified in the focus groups described below (Phase 2).

### Phase 2: conduct MOVE ON focus groups to identify unique barriers to behaviour change

Front-line clinicians from each hospital site were invited to participate in focus group sessions, prior to implementing the MOVE ON intervention, through email invitations, posters on the unit, and one-on-one communication from unit managers. Eligible participants included nurses, nurse practitioners, occupational therapists, physiotherapists, physicians, managers, other allied health-care staff, and other unit staff such as personal support workers and ward clerks. The focus groups, which were conducted at each of the hospitals, were moderated by each site’s education coordinator and/or a research coordinator. Training and coaching in focus group methodology was provided to personnel at each site as needed. A semi-structured focus group protocol (see Additional file 1)—developed by the central MOVE ON Team—was used to assess clinicians’ knowledge about mobilization, the factors they perceived as facilitators and barriers to mobilization, and their capability and readiness to implement an early mobilization strategy. Facilitators probed participants to consider the underlying factors related to suggested barriers. Participants were then presented with the three recommendations of MOVE ON and asked to propose strategies and suggestions for how such an initiative could be implemented and sustained within the local context.

Focus groups were audio recorded and transcribed verbatim. The transcripts were analyzed by two qualitative analysts working with the central MOVE ON Team. The analysts used a thematic analysis approach [25], first familiarizing themselves with the data to develop initial coding categories and then refining the categories into subcategories to form patterns and themes that were ultimately used to develop a thematic framework. All transcripts were independently coded by two research associates using NVivo 10 software (QSR International, Cambridge, MA). Each site was given a report with the aggregated qualitative findings from all of the MOVE ON focus groups.

### Phase 3: collect data on adaptations after implementation of MOVE ON

Each hospital was given an intervention activity tracking sheet and asked to record all of the activities introduced to their units, including those chosen from the intervention ‘menu’ and those developed on site. These sheets were collated by the central MOVE ON Team. Hospitals were also asked to provide descriptions of all activities, roles, the number of staff attended, and when they occurred.

### Phase 4: map barriers and intervention activities to the COM-B system

Using data from the focus groups and the list of tailored intervention activities implemented at each hospital, the COM-B system was used as a taxonomy to classify barriers to behaviour change (as identified during the focus groups) and to classify the hospitals’ intervention activities.

More specifically, two reviewers from the central MOVE ON Team independently classified each barrier under relevant categories of the COM-B (capability, motivation, or opportunity), applying more than one category if appropriate. The same exercise was conducted separately for the list of intervention activities. Inter-rater reliability (i.e. the degree of agreement between the two reviewers) was compared by calculating percent agreement, and any discrepancies were reconciled through discussion. The results were compiled and sent to the central MOVE ON Team for review and feedback.

### Phase 5: develop a mapping guide for MOVE ON + adaptations

The results of Phases 1 through 4 were used to enhance the implementation of interventions, in preparation for the MOVE ON + stage of the project. Specifically, after the central MOVE ON Team had approved the classifications of barriers and intervention activities, COM-B categories were used to match barriers to corresponding intervention activities in the same category.

## Results

### Phase 1: menu of suggested intervention activities based on MOVE iT data

A menu of 14 suggested intervention activities (Table 1) was provided to all participating hospitals. The hospitals used many of these interventions (as described below) and also created their own.

**Table 1:** **Menu of suggested interventions**

**Intervention type**	**Activity**	**Description**
Educational meetings (in person or electronic)	Inter-professional staff education module^a^	Classroom-based module to help prepare staff for a change in clinical practice related to mobilization by facilitating discussion and acknowledging potential challenges and barriers in a group setting
	MOVE iT/MOVE ON mobilization of vulnerable elders in Ontario electronic module^a^	Electronic module used to quickly reach a large number of staff, with content similar to that of the classroom-based module
	MOVE ON senior-friendly hospital module^a^	Electronic module for inter-professional hospital staff to review the risks of hospitalization for older adults, reflect on the effects of ageism and stereotyping of older adults, and review the needs of older adults
	Clinical coaching	Education coordinators provided bedside coaching with staff to walk through potential ways to mobilize patients in different scenarios
One-on-one staff coaching tools	Review of ABC educational tool	One-on-one staff coaching tool^b^ to review the standard of care for mobility
	Documentation practices	One-on-one staff coaching tool^b^ to encourage proper documentation of patients’ mobility status
	Transfer techniques and ergonomics education sessions	One-on-one staff coaching tool^b^ presenting techniques such as ‘roll’, ‘lie to sit’, and ‘sit to stand’
	Natural opportunities	One-on-one staff coaching tool^a^ to encourage creative ways to incorporate mobility into everyday practice
Distribution of printed educational materials	Mobility algorithm	Tool to help staff assess each patient’s mobility status and to aid in communicating patients’ mobility status through the use of (ABC) letters to identify mobility level
	Hazards of immobility poster	Educational poster for hospital staff
	Benefits of getting out of bed while in hospital poster	Educational poster for patients/family members
	Keep moving pamphlet	Educational pamphlet for patients/family members
Reminders	Commercial breaks	One-minute musical interludes during multidisciplinary or bullet rounds with messages to encourage mobilization
Communication and case discussion	Huddles	Quick stand-up staff meetings to discuss progress of intervention and share successes and challenges
Educational exhibits	MOVE ON fair	Series of eight stations set up in a common area on at least two separate days, where staff members can learn about MOVE ON, documentation practices, myths about mobilization, and other relevant aspects of the project

^a^Although staff education was mandatory, the mode of education was at the discretion of each site.

^b^Knowledge-to-practice coaching is delivered at the point of care. It can be used to engage staff on an individual basis to support mobility coaching by the bedside and relates knowledge and skills directly to patients that the staff member is caring for that day.

### Phase 2: barriers to behaviour change identified by focus groups

All 14 hospitals conducted on-site focus groups with their staff. Participating staff members included nurses, nurse practitioners, occupational therapists, physiotherapists, physicians, managers, various allied health staff, and other unit staff such as personal support workers and ward clerks. The number of participating units per hospital ranged from 1 to 4. Up to 12 focus groups were conducted at each hospital, with the total number of participants per hospital ranging from 5 to 81 (Table 2).

**Table 2:** **Participation in focus groups by hospital sites**

**Hospital**	**Number of participating patient care units (** ***n*** **=26)**	**Number of focus groups (** ***n*** **=46)**	**Total number of participants (** ***n*** **=261)**
A	2	2	22
B	3	3	8
C	3	4	22
D	1	1	9
E	3	2	13
F	2	1	5
G	2	2	11
H	2	4	22
I	2	3	24
J	2	3	15
K	2	12	81
L	2	2	13
M	2	2	7

The barriers to behaviour change identified during the focus groups can be conceptualized at three different levels: the health-care provider, the patient, and the hospital unit (see Additional file 2 for an overview of the results). To understand the root causes of barriers, during the coding phase, analysts coded barriers into larger themes to understand the commonalities across sites.

There were several barriers identified at the health-care provider level, such as lack of knowledge about the benefits and importance of early mobilization, attitudes and beliefs about mobilization, a perceived lack of skills to implement the MOVE ON intervention, and fear of injuring the patient. However, time constraints and heavy workload were the most commonly mentioned barriers by health-care providers, who perceived that these factors would prevent them from spending the time needed to mobilize patients. Others expressed resistance towards implementing the intervention because they did not perceive mobility as their responsibility and felt it would add to their already heavy workload. In addition, several health-care provider participants mentioned lack of clarity about staff roles and responsibilities as a barrier to implementing an early mobilization strategy. Many staff members indicated that they usually have no knowledge about the patients’ baseline mobility status and as such are hesitant to initiate any mobility activities.

At the patient level, participants mentioned barriers such as attitudes and beliefs about mobilization on the part of both patients and their families. Many clinicians described patients portraying a ‘sick role’, with the accompanying misconception that while they are in the hospital it was safer for them to stay in bed. Focus group participants perceived this belief as also being held by families and caregivers, often contrary to the advice of health-care providers. Other barriers participants faced while assisting patients to mobilize included patients’ physical and cognitive status and lack of personal mobility aids present during hospital stay.

Lack of resources was one of the most commonly mentioned barriers to implementing the MOVE ON interventions identified at the level of the participating hospital unit. In particular, some hospital units lacked relevant mobility equipment, space for mobility exercises, or a consistent system to document and monitor mobility. In addition, many units recognized the need for more support staff to encourage mobilization on weekends, such as personal care assistants, physiotherapy assistants, and allied health professionals. Other unit barriers, unrelated to resource issues, included competing quality improvement initiatives and other priorities, as well as lack of accountability for mobility among unit staff.

### Phase 3: adaptations during implementation of MOVE ON

Participating hospitals implemented various combinations of the 14 intervention activities (Table 1) in their units. As required, all sites implemented education-based activities such as classroom and electronic modules across the unit. Additional tailored activities developed by the participating units included documentation-related activities, appointment of mobility champions, and distribution of promotional materials and reminders for staff. One example of a documentation activity was the ‘mobility clock’, a visual tool in the form of a paper clock on which the arms were positioned to indicate the patient’s mobility status. The mobility clock was posted above each patient’s bed and used as a mechanism for communication among staff members, patients, and their families. The arms could be adjusted by staff according to the patient’s mobility progress. The use of promotional materials, including newsletter items, was an activity selected to increase awareness of the MOVE ON intervention. The list of additional intervention activities and their descriptions are provided in Additional file 3.

### Phase 4: barriers and intervention activities mapped to the COM-B system

There was excellent agreement between the reviewers (AM and JEM) who independently categorized the barriers and intervention activities (using the COM-B framework (88% agreement for both barriers and facilitators). The only discrepancies occurred in cases where an item was classified into two or more COM-B categories. In these cases, the two reviewers agreed for at least one category but differed on the second category. For example, both reviewers classified documentation-related activities, such as the mobility clock and the whiteboard, as ‘opportunities’, because these tools create a physical opportunity to perform a specific behaviour (i.e. documenting the patient’s mobility status). Whereas one of the reviewers also initially classified these activities under ‘motivation’, the second reviewer did not. Five such discrepancies were reconciled through discussion, and the results (Additional file 4) were discussed by the central MOVE ON Team.

### Phase 5: mapping guide for MOVE ON + adaptations

The results of the mapping exercise in Phase 4 were used to develop a mapping guide, a quick reference for identifying appropriate strategies to meet identified barriers (Table 3). Within the guide, each barrier is listed under one or more of the COM-B categories. For example, ‘existing climate/culture of unit’ is listed under both ‘opportunity’ and ‘motivation’ because the social environment within a hospital unit can dictate whether there are opportunities for teamwork or support from management and also whether staff will have the motivation to participate in an early mobilization strategy. To target this barrier, intervention activities that increase staff collaboration and incorporate mobilization as part of the unit’s cultural milieu can be considered. Huddles are an example of an activity that creates the opportunity for staff to communicate and support each other in carrying out the intervention. Mobility champions can provide role modelling for staff and can motivate staff to get involved in safe and effective mobilization of patients.

**Table 3:** **Reference guide for mapping barriers with appropriate intervention activities**

	**Barriers**	**Intervention activities**
Capability	• Attitudes and beliefs about mobilization	• Classroom education
	• Lack of knowledge about the importance of mobilization	• Follow-up education (e.g. one-on-one coaching)
	• Perceived lack of skills to implement intervention	• Staff and patient posters
	• Fear of injuring patient	• Patient pamphlets/handouts
	• Little to no knowledge of patient’s baseline or current mobility status	• Display
	• Patient/family beliefs about mobilization	• Promotions
		• Seniors’ fair (contest)
		• Volunteer activities
Opportunity	• Time constraints and heavy workload	• Leadership activities
	• Lack of clarity regarding roles and responsibilities	• Huddles
	• Lack of standard mobility documentation processes	• Staff meeting/rounds
	• Presence of other priorities and initiatives on the unit	• Promotions
	• Existing climate/culture of unit	• Reminders
	• Lack of communication between health-care providers regarding patient’s care plan	• Mobility champions
	• Patient lack of personal mobility aids	• Volunteer activities
	• Lack of resources	• Documentation
	• Lack of accountability	• Equipment
	• Patient’s acuity	
Motivation	• Attitudes and beliefs about mobilization	• Reminders
	• Resistance to implement intervention	• Follow-up education (i.e. one-on-one coaching)
	• Lack of clarity regarding roles and responsibilities	• Mobility champions
	• Existing climate/culture of unit	• Audits
	• Lack of accountability	• Documentation
	• Patient/family beliefs about mobilization	• Leadership activities
	• Patient lack of motivation	• Patient social activities
		• Volunteer activities

The central MOVE ON Team will use the guide (Table 3) to facilitate the MOVE ON + project, an expansion of MOVE ON. During the MOVE ON + focus group sessions, each site will participate in a barrier ranking exercise (rather than simply listing perceived barriers and facilitators). The information collected will be used to generate a tailored list of intervention activities and suggested adaptations for each specific site according to their prioritized barriers and facilitators.

## Discussion

The current project used behaviour change theory in conjunction with a real-world implementation intervention to develop a systematic method for adapting intervention activities. Through focus groups with 261 front-line clinicians across 14 hospitals, we identified barriers to behaviour change at three levels: health-care providers, patients, and hospital units. We then documented all implementation activities that were delivered across sites. The barriers and implementation activities were mapped onto the COM-B behaviour change theory with a high level of agreement between raters (88%). Results of the mapping exercise were used to create a mapping guide to assist future sites in selecting intervention activities that appropriately target the underlying behaviour change construct of identified barriers.

Based on the focus group findings, time constraints and heavy workloads were perceived as significant barriers preventing health-care providers from mobilizing their patients. Focus group participants also felt that patients’ perceptions of mobilization and the desire to remain sedentary while sick (termed ‘the sick role’) were common patient-level barriers to mobilization, and many participants described struggling with a lack of proper equipment and human resources needed to implement the actionable recommendations of the intervention. Previous studies have described similar barriers to implementation of interventions involving care of elderly patients [26,27] and various health-care providers, such as nurses and physicians [28-32]. The identification and listing of barriers is not a novel undertaking on its own; however, the analysis of these barriers across multiple settings that implemented an early mobilization strategy and using them to develop an implementation guide is unique and has the potential to impact implementation practices across multiple health outcomes.

Interventions are more likely to influence change if they are tailored to target the factors underlying barriers to behaviour change [33]. For example, the staff from the majority of MOVE ON sites stated that health-care providers’ attitudes and beliefs about mobilization, and resistance to implementing interventions were significant barriers to implementation of the MOVE ON intervention. However, before an intervention activity is selected or modified to address these barriers, it is important to consider the root of the barrier. For this study, understanding root causes was done on two levels: during focus groups and during qualitative coding. Through focus group discussions, participants were asked to provide feedback on their initial thoughts about barriers; facilitators then probed participants to consider the underlying factors related to each barrier. During data analysis, these barriers were coded into larger themes (e.g. ‘time constraints’ were coded as a resource issue) to understand the common threads across sites. In the current example, was it a lack of awareness or just an indifference that is influencing health-care providers’ attitudes and beliefs about mobilization? Similarly, resistance to implementation may be related to a lack of information and skills or a personal apathy towards patient mobilization. In order to make changes in clinical practice, strategies to address barriers must consider underlying causes and must be multi-faceted as well as evidence-based [13]. To address the factors described above, intervention activities are needed that will motivate staff to become involved in the MOVE ON intervention and to mobilize their patients. One example of such an intervention activity is the use of ‘mobility champions’, staff members who act as role models or mentors, encouraging, carrying out, and facilitating the safe and effective mobilization of patients. They demonstrate exemplary willingness, understanding, and determination in helping to reduce patient inactivity and promote staff involvement in routine mobilization of patients. A systematic review of the diffusion of innovations in service organizations has shown that individuals are more likely to adopt an intervention if key individuals in their social networks are willing to support the initiative [34-36]. Another intervention activity targeting the motivation of health-care providers is the use of leadership initiatives. At some MOVE ON sites, management has incorporated mobility measures into managerial leadership evaluation measurement goals or decision support bi-annual surveys. There is literature that confirms that if leadership demonstrates strong support for an intervention and its messages, they are more likely to enhance the implementation process [34,37-39].

The main advantage of the mapping guide presented here is that it provides a schema through which implementers can easily identify potential strategies to overcome barriers to implementation at the local level. This is particularly useful when an intervention is being spread and scaled up across multiple units and hospitals. A second advantage of a systematic approach to intervention adaptations is that decisions about adaptations can be documented and included in future analyses when outcomes of the intervention are being evaluated. The current implementation literature lacks information about the effects of specific adaptations on program outcomes [16]. This lack of information has played a role in an ongoing debate about the importance of fidelity to the original design of an intervention versus the importance of adapting interventions to the local context [14,40]. In the next phase of our initiative, participating sites will use the results from Table 3 to map the interventions to facilitate early mobilization of older adults. Given the scarce resources, competing demands, and a large number of barriers, prioritization of barriers and intervention activities is critical to effectively implement intervention. For the MOVE ON + intervention, focus group participants will be asked to rank barriers in order to prioritize intervention activities. Since we have developed a large pool of barriers, they will be asked to select the most significant barriers. Once barriers have been prioritized, they will use the mapping guide to select appropriate intervention activities.

Some limitations to the approach described in this study should be noted. First, the perspective of elderly patients and their families was not included. These groups are important stakeholders in the implementation of MOVE ON, and there may have been additional information on barriers which was not taken into account; therefore, the barriers included in the mapping guide are only from the health-care provider perspective. However, surveys were conducted post implementation of MOVE ON but no new themes on barriers were revealed. Second, although there were a range of inter-professional staff involved in the focus groups overall, recruitment was challenging, and some focus groups contained only one type of health professional (i.e. six nurses in one focus group, five physiotherapists, etc.). It may have been beneficial to have the perspectives of more allied health staff such as physiotherapy assistants, dietitians, and social workers. Third, the nature of focus group data is that it contains the self-reported perceptions of barriers to implementation. In order to gather additional context for implementation at each unit, it may have been useful to have collected observational data, collected by an external MOVE ON Team member, on barriers to implementation as well as on intervention adaptations. All the data collected for the development of this guide was self-reported by hospital unit staff. Fourth, our approach is for the implementation of an intervention within a hospital setting. It is possible that the mapping guide does not include certain other previously reported barriers that are common in other health-care settings, such as walk-in clinics, community health centres, long-term care centres, or family health teams. Those wishing to use a similar systematic approach to adaptations would need to first perform a literature scan for additional barriers associated within other health-care settings.

## Conclusions

A systematic guide for adapting an intervention in multiple sites was developed and will be used for further spread of an early mobilization intervention for older adults admitted to hospital. This mapping guide allows implementers to identify potential implementation strategies that will overcome local barriers, thereby enhancing implementation.

## Additional files

Additional file 1:
**Focus group protocol.** A semi-structured focus group protocol—developed by the central MOVE ON Team—which was used to assess clinicians’ knowledge about mobilization, the factors they perceived as facilitators and barriers to mobilization, and their capability and readiness to implement an early mobilization strategy. Additional file 2:
**Barriers to implementation.** An overview of barriers to implementation identified during the focus groups which are conceptualized at three different levels: the health-care provider, the patient, and the hospital unit. Additional file 3:
**Intervention activities and adaptations.** A list of intervention activities and adaptations described by hospital units. Additional file 4:
**Final results of mapping exercises.** Barriers and intervention activities mapped to the COM-B system.

## Abbreviations

COM-B framework: Capability, opportunity, motivation-behaviour framework
HCP: Health-care provider
MOVE ON: Mobilization of Vulnerable Elders in Ontario
MOVE iT: Mobilization of Vulnerable Elders in Toronto
