# Supplementary material for: Mapping barriers and intervention activities to behaviour change theory for Mobilization of Vulnerable Elders in Ontario (MOVE ON), a multi-site implementation intervention in acute care hospitals
Source: Implement Sci. 2014 Oct 30;9:160. doi: 10.1186/s13012-014-0160-6 (PMC4225038; doi:10.1186/s13012-014-0160-6)
Supplement: Additional file 1: — Focus group protocol. A semi-structured focus group protocol—developed by the central MOVE ON Team—which was used to assess clinicians’ knowledge about mobilization, the factors they perceived as facilitators and barriers to mobilization, and their capability and readiness to implement an early mobilization strategy. [file 13012_2014_160_MOESM1_ESM.docx]

**Additional file 1. Focus Group Protocol**

Process for Focus Groups

1. Introductions (consent obtained)

2. Review of purpose of meeting

**Part 1**

1. Do you think there is sufficient evidence to implement an early mobilization strategy?

a. If not, what more is needed?

2. Who should be involved in an early mobilization strategy targeting internal medicine patients at your institution?

a. Who are the key stakeholders?

b. How should they be engaged?

3. Do you feel you would be able to implement an early mobilization strategy?

4. What resources would you need?

a. Financial

b. Human

c. Environmental

d. Other?

**Part 2**

Goals of an early mobilization strategy would focus on actionable recommendations:

1) Encourage mobilization at least three times a day

2) Mobilization should be progressive and scaled

3) Mobility assessments should be implemented within 24 hours of the decision to admit

How would you suggest these recommendations be implemented?

a. What strategies would you recommend?

b. Who should be targeted for each?

c. How would you measure impact?

**Part 3**

The MOVE iTgroup has developed educational modules, a care algorithm, patient poster – the

interviewer will show each of these and ask for feedback on each tool individually.

• Would this tool help you?

• What information is missing?

• Is there any material that needs clarification?
