# Supplementary material for: Mapping barriers and intervention activities to behaviour change theory for Mobilization of Vulnerable Elders in Ontario (MOVE ON), a multi-site implementation intervention in acute care hospitals
Source: Implement Sci. 2014 Oct 30;9:160. doi: 10.1186/s13012-014-0160-6 (PMC4225038; doi:10.1186/s13012-014-0160-6)
Supplement: Additional file 2: — Barriers to implementation. An overview of barriers to implementation identified during the focus groups which are conceptualized at three different levels: the health-care provider, the patient, and the hospital unit. [file 13012_2014_160_MOESM2_ESM.docx]

**Additional file 2. Barriers to Implementation**

|  | **Perceived Barriers** |
| --- | --- |
| Patient level | - Patient’s acuity makes mobilization difficult (dementia/delirium, physical weight etc.) - Patients have little knowledge of the importance of early mobility - Patients lack motivation to move (e.g., depression) - Patients do not cooperate with instructions to mobilize - Patients have a belief that they are too sick to a participate in mobility activities (i.e., the ‘sick role’) - Patients have a fear of falling - Patients are missing their personal mobility aids from home |
| Health care provider level | - Providers have little to no knowledge of patient’s baseline or current mobility status - There is staff resistance to implementation due to: (1) lack of knowledge regarding mobilization techniques; (2) view mobility care as not their responsibility; and (3) fears of injuries to patient or themselves - Lack of consistency and communication between health care providers regarding patient’s care plan and the importance of mobility - Heavy workload and lack of time prevent staff from regularly mobilizing patients |
| Unit level | - Lack of the following resources:  1. Staff    - 1. Lack of Physiotherapists on weekends      2. Lack of Physiotherapist assistants      3. Lack of personal care assistants (PCAs)      4. Lack of volunteers to assist in regularly mobilizing patients 2. Equipment    - 1. Lack of proper mobility equipment (ex. Chairs, Lifts)      2. Lack of system to monitor and maintain equipment in the unit 3. Space    - 1. Rooms, hallways, and units not properly designed to promote mobility  - Competing unit priorities (other mobility initiatives or programs occurring simultaneously) - Lack of consistent system to document and monitor mobility - Placement of patient care equipment/support (e.g., lines, tethers) - Lack of accountability for staff implementing the actionable recommendations |
