# Supplementary material for: Mapping barriers and intervention activities to behaviour change theory for Mobilization of Vulnerable Elders in Ontario (MOVE ON), a multi-site implementation intervention in acute care hospitals
Source: Implement Sci. 2014 Oct 30;9:160. doi: 10.1186/s13012-014-0160-6 (PMC4225038; doi:10.1186/s13012-014-0160-6)
Supplement: Additional file 3: — Intervention activities and adaptations. A list of intervention activities and adaptations described by hospital units. [file 13012_2014_160_MOESM3_ESM.docx]

**Additional file 3. Intervention Activities & Adaptations**

| **Intervention Type** | **Activity** | **Description of Intervention Activity** |
| --- | --- | --- |
| **Educational Meetings** | **Classroom education (P- in person or E- electronic)** | Education sessions that help prepare staff for a change in clinical practice by facilitating discussion and acknowledging potential challenges and barriers to implementing the mobilization intervention. These sessions can be delivered in person, as well as over the web. |
|  | **Follow-up education (i.e. one-on-one coaching)** | Knowledge-to-practice coaching that is delivered at the point-of-care. Such that education coordinators or physiotherapists teach mobilization strategies to staff on an individual basis at patient's bed-sides; and relates the knowledge and skills directly to the patients being cared for that day. |
|  | **Grand rounds/Presentations** | Educational rounds that provide an opportunity for staff to learn and discuss various topics related to clinical practice. In some occasions, mobilization is the topic of focus. |
| **Distribution of printed educational materials** | **Promotions (i.e. newsletters, email blast, commercial break, promoting mobility wheel (via email/visits))** | Activities that increase awareness of mobilization activities in the hospital and community. These can include blurbs in staff newsletters/newspapers, announcements, star magnets, large banners, buttons and email blast. |
|  | **Staff posters** | Posters that illustrate strategies staff can use to mobilize patients and the hazards of immobility for older patients. These can include posters that display the mobilization algorithm or outline the biological and psychological effects of immobility. |
|  | **Patient Posters** | Posters that illustrate the benefits of and strategies for mobilization during patient's hospital stay. These can include posters that outline the biological, psychological or social benefits of mobilizing during a patient's hospital stay, as well as displays that describe techniques patients can use to stay active during their hospital stay. |
|  | **Patient pamphlets/handouts** | Information booklets that are given to patients at discharge or during their hospital stay. They contain facts and strategies for mobilization. |
|  | **Mobility Algorithm** | A step-by-step procedure for clinicians to assess a patient’s level of mobility. |
| **Reminders** | **Visual Facts/Tips** | Visual display that uses daily or weekly updated facts, statistics or recommendations from research articles to prompt staff to carry out mobilization activities. Ex., daily tips. |
| **Communication and case discussion** | **Huddles** | Quick meeting that pulls staff and team members together to discuss improvement opportunities (i.e., project progress and successes or challenges related to mobilization activities). |
|  | **Nurse/staff bullet rounds** | Very brief discussions between staff regarding pressing issues concerning patient's medical status and mobility requirements. |
|  | **Staff meeting/rounds** | Staff members meet to update and discuss their issues with regards to the mobilization intervention and general hospital functioning. Some topics under discussion can include: physiotherapists role, mobility champions, and motivational speeches. |
| **Educational Exhibits** | **Seniors’ fair (contest)** | A staff member fair and/or contest that displays a variety of exhibits that share information and pictures about MOVE ON. |
|  | **Visual Display** | Exhibit that displays videos and animated objects (i.e. snowman) for viewing by the public (i.e. staff, patients, community). The two minute video demonstrates a physiotherapist performing a transfer with a patient while describing proper techniques. |
| **Provider Incentives** | **Management leadership activities** | Senior management's activities that supported the implementation of MOVE ON activities. These can include the incorporation of mobility measures into managerial Leadership Evaluation Measurement goals or Decision Support biannual rounding surveys. As well as providing additional resources, such as chair lifts, transfer equipment and documentation. |
|  | **Equipment (chair alarms)** | Alarms attached to wheel chairs that alert staff when patients are trying to stand. Helps to promote safety. |
| **Patient Interventions** | **Patient's social motivation** | Social activities that help to increase patient's motivation to mobilize. Ex., marked meters in the hallway that patients can use to track the distance they have walked, morning/evening exercise classes, eating dinner/lunch in communal dinning/lunch room. |
|  | **Volunteer activities** | Hospital units engage volunteers during project implementation by having the volunteers emphasize the importance of mobility for elderly patients, assist with observation walks and restock patient/staff posters. |
| **Local Opinion Leaders** | **Mobility champions** | Are role-models and/or mentors who encourage, carry out and facilitate the safe and effective mobilization of patients. They demonstrate willingness, understanding, and a determination in helping to reduce patient inactivity and promote staff involvement in the routine mobilizing of patients. |
| **Audit & Feedback** | **Unit Staff Audits** | Visual audits and feedback given to staff weekly |
| **Staff Organization** | **Documentation (i.e. whiteboard, mobility wheel, log sheet)** | Techniques for tracking patient’s mobility status. These include: (a) whiteboard– in-room message board displaying patient specific information including mobility status; (b) 24 Hour Flow sheet– a comprehensive log of the patient’s medical indicators and information describing patient’s activity status and frequency of activities of daily living measures; (c) Mobility assessment sheet– a log of patient’s activity level and capability; (d) Mobility magnets– used to indicate mobility level. |
