# Supplementary material for: Mapping barriers and intervention activities to behaviour change theory for Mobilization of Vulnerable Elders in Ontario (MOVE ON), a multi-site implementation intervention in acute care hospitals
Source: Implement Sci. 2014 Oct 30;9:160. doi: 10.1186/s13012-014-0160-6 (PMC4225038; doi:10.1186/s13012-014-0160-6)
Supplement: Additional file 4: — Final results of mapping exercises. Barriers and intervention activities mapped to the COM-B system. [file 13012_2014_160_MOESM4_ESM.docx]

**Additional file 4. Final Results of Mapping Exercises**

| **Barrier** | **COM-B Factor** | **COM-B Factor** |
| --- | --- | --- |
| **Attitudes and Beliefs about Mobilization** | Capability | Motivation |
| **Lack of Knowledge about the importance of Mobilization** | Capability |  |
| **Little to no knowledge of patient's baseline or current mobility status** | Capability |  |
| **Patient's Acuity** | Opportunity |  |
| **Perceived Lack of Skills to Implement Intervention** | Capability |  |
| **Time Constraints and Heavy Workload** | Opportunity |  |
| **Resistance to implement intervention** | Motivation |  |
| **Lack of Clarity Regarding Roles and Responsibilities** | Opportunity | Motivation |
| **Lack of Standard Mobility Documentation Processes** | Opportunity |  |
| **Presences of other priorities and initiatives on the unit** | Opportunity |  |
| **Existing Climate/Culture of unit** | Opportunity | Motivation |
| **Patient/Family beliefs about mobilization** | Capability | Motivation |
| **Patient lack of personal mobility aids** | Opportunity |  |
| **Patient lack of motivation** | Motivation |  |
| **Fear of injuring patient** | Capability |  |
| **Lack of communication between health care providers regarding patient's care plan** | Opportunity |  |
| **Lack of resources** | Opportunity |  |
| **Lack of accountability** | Opportunity | Motivation |

| **Intervention Activity** | **COM-B Factor** | **COM-B Factor** | **COM-B Factor** |
| --- | --- | --- | --- |
| **Huddles** | Opportunity |  |  |
| **Seniors fair (contest)** | Capability |  |  |
| **Display** | Capability |  |  |
| **Staff meeting/rounds** | Opportunity |  |  |
| **Promotions (i.e. newsletters, email blast, commercial break, promoting mobility wheel (via email/visits))** | Capability | Opportunity | Capability |
| **Staff posters** | Capability |  |  |
| **Patient Posters** | Capability |  |  |
| **Patient pamphlets/handouts** | Capability |  |  |
| **Nurse/staff bullet rounds** | Opportunity |  |  |
| **Reminders** | Motivation | Opportunity |  |
| **Classroom education (P- in person or E- electronic)** | Capability |  |  |
| **Follow-up education (i.e. one-on-one coaching)** | Capability |  |  |
| **Mobility champions** | Opportunity | Motivation |  |
| **Volunteer activities** | Opportunity | Motivation | Capability |
| **Audits** | Motivation |  |  |
| **Documentation (i.e. whiteboard, mobility wheel, log sheet)** | Opportunity | Motivation |  |
| **Equipment** | Opportunity |  |  |
| **Patient's social motivation** | Motivation |  |  |
| **Grand rounds/Presentations** | Capability |  |  |
| **Leadership activities** | Opportunity | Motivation |  |
